# Interdisciplinary and interprofessional communication intervention: How psychological safety fosters communication and increases patient safety

**DOI:** 10.3389/fpsyg.2023.1164288

**Published:** 2023-06-15

**Authors:** Johanna Elisa Dietl, Christina Derksen, Franziska Maria Keller, Sonia Lippke

**Affiliations:** ^1^Health Psychology and Behavioral Medicine, School of Business, Social and Decision Science, Constructor University, Bremen, Germany; ^2^Klinikum Bremerhaven Reinkenheide gGmbH, Treatment Center for Psychiatry, Psychotherapy and Psychosomatic, Bremerhaven, Germany

**Keywords:** healthcare, communication intervention, interdisciplinary teams, psychological safety, patient safety

## Abstract

**Background:**

Effective teamwork and communication are imperative for patient safety and quality care. Communication errors and human failures are considered the main source of patient harm. Thus, team trainings focusing on communication and creating psychologically safe environments are required. This can facilitate challenging communication and teamwork scenarios, prevent patient safety risks, and increase team performance perception. The sparse research concerning communication interventions calls for an understanding of psychological mechanisms. Therefore, this study investigated mechanisms of an interpersonal team intervention targeting communication and the relation of psychological safety to patient safety and team performance perception based on the applied input–process–output model of team effectiveness.

**Methods:**

Before and after a 4-h communication intervention for multidisciplinary teams, a paper–pencil survey with *N* = 137 healthcare workers from obstetric units of two university hospitals was conducted. Changes after the intervention in perceived communication, patient safety risks, and team performance perception were analyzed *via t-*tests. To examine psychological mechanisms regarding psychological safety and communication behavior, mediation analyses were conducted.

**Results:**

On average, perceived patient safety risks were lower after the intervention than before the intervention (*M*_*T*1_ = 3.220, *SD*_*T*1_ = 0.735; *M*_*T*2_ = 2.887, *SD*_*T*2_ = 0.902). This change was statistically significant (*t* (67) = 2.760, *p* =.007). However, no such effect was found for interpersonal communication and team performance perception. The results illustrate the mediating role of interpersonal communication between psychological safety and safety performances operationalized as perceived patient safety risks ($\alpha_{1}^{{}^{*}}\beta_{1}$ = −0.163, 95% CI [−0.310, −0.046]) and team performance perception ($\alpha_{1}^{{}^{*}}\beta_{1}$ = 0.189, 95% CI [0.044, 0.370]).

**Discussion:**

This study demonstrates the psychological mechanisms of communication team training to foster safety performances and psychological safety as an important predecessor for interpersonal communication. Our results highlight the importance of teamwork for patient safety. Interpersonal and interprofessional team training represents a novel approach as it empirically brings together interpersonal communication and collaboration in the context of patient safety. Future research should work on follow-up measures in randomized-controlled trials to broaden an understanding of changes over time.

## 1. Introduction

Progressive complexity and high demands prevent high-*quality care and patient safety* in various healthcare contexts. To meet these demands, effective teamwork and communication are key values to deliver high-quality care (Knox and Simpson, 2011; Weller et al., 2014; Rosen et al., 2018). While functioning teamwork is associated with quality of care and patient safety, communication and teamwork failures in interdisciplinary teams lead to deficient patient care and thus pose safety risks (Weller et al., 2014; Rosen et al., 2018; Parker et al., 2019).

According to the literature, patient safety is defined as the absence of *preventable adverse events* (PAEs) that are caused by care below existing standards rather than a patient's condition itself (Griffin and Resar, 2009). This definition illustrates the duties of healthcare organizations and healthcare workers (HCWs), which are responsible for the prevention, reduction, and learning of failure and adverse events to improve safety and care delivery. PAEs are defined as unintentional harm which arises from deficits in the collaboration of healthcare providers (Mitchell, 2008). Hence, patient safety and high-quality care depend on interdisciplinary *teamwork* (Manser, 2009). HCWs must adapt to complex environments such as rapidly changing circumstances, patient conditions, fast knowledge, technology development, and team compositions (Rosen et al., 2018). Furthermore, team learning is especially important in healthcare because it is highly interdependent; hence, HCWs must rely on other interdisciplinary and interprofessional team members to combine and apply specific job-related knowledge and skills for better patient care (Derickson et al., 2015). Therefore, to *collaborate and communicate* is crucial to provide patient care and patient safety (Manser, 2009).

The detection and communication of adverse events in healthcare are essential for patient safety. A thorough application of incident reporting systems is driving failure learning behavior since they provide information, progress safety, and hold HCWs responsible for their performance. Error reporting benefits suggestions for decreasing and eliminating errors (Kohn et al., 2000). Nevertheless, adverse events are often underreported in healthcare settings due to a variety of factors, including fear of retribution, blame, job loss, lack of knowledge or awareness, concerns about legal liability, reputation harm, feelings of guilt, inadequate training, or different understanding of error detection (Evans et al., 2006; Stewart et al., 2018). Furthermore, there are data collection challenges on adverse events as it is time-consuming and resource-intensive or may not capture all types of errors (Evans et al., 2006; Kaldjian et al., 2008; Unal and Seren, 2016). The information majority on the frequency of PAEs derives from case studies, retrospective reviews, patient records, or formal incident reporting. While these documentations and analyses of PAEs indicate the occurred PAE or adverse consequences, they rarely focus on their potential processes or triggers (Forster et al., 2006; Keller et al., 2021). Therefore, the existing error tools are not always suitable for drawing conclusions about errors or error experiences. Moreover, assessments of teamwork factors can be impractical or difficult to implement; thus, (team) perceptions are a capable resource to achieve teamwork insights (Mathieu et al., 2008; Kämmer et al., 2023), which applies especially to stressful obstetric processes. Therefore, assessing perceived patient safety risk triggers or perceived team performance perception from a HCWs' perspective is an appropriate approach.

*Interpersonal communication* in healthcare is described as an interactive exchange process, to achieve a common understanding between HCWs within the team as well as between patients and providers (Echterhoff et al., 2009). Communication is shown to be the primary root cause of serious patient harm as it is a significant contributor to healthcare errors. Risk factors of communication deficits, which contribute to poor patient experience and thus impact patient safety, are the lack of effective handovers, inaccurate diagnosis, treatment errors, and inefficient patient–provider and team interaction. Thus, poor interpersonal communication may lead to weak performance, patient injury, or even death (Gluyas, 2015; Foronda et al., 2016; Burgener, 2017; Bekkink et al., 2018). Furthermore, flawed safety cultures in hierarchically structured hospitals hinder communication by inhibiting speaking up behavior (Nembhard and Edmondson, 2011), showing how closely effective communication is interrelated with teamwork and safety performance.

Previous evidence has identified *psychological safety* as an important factor supporting communication and teamwork in healthcare. It describes team members' level of feeling safe to take interpersonal risks (Derickson et al., 2015). Psychological safety is associated with patient safety, collaboration, involvement in quality improvement work, learning from mistakes, and adverse events (Hirak et al., 2012; Arnetz et al., 2019), which indicates the connection between communication, safety performance, and environments that are perceived as safe (similar as psychological safety). Consequently, fostering a culture of openness around error reporting is essential to increase patient safety (WHO, 2019). This can be achieved through training regarding communication and teamwork (Ito et al., 2022). These factors are promoted by creating an atmosphere of psychological safety in healthcare settings, which in turn leads to more interpersonal communication and knowledge sharing (Leroy et al., 2012; Newman et al., 2017). Previous research has found that psychological safety is linked to several communication outcomes, such as speaking up behavior or reduction in silence behavior (Newman et al., 2017). Moreover, psychological safety can be regarded as a team attribute or process promoting input by feeling safe for engaging in risk interaction with colleagues (Haviland et al., 2022). Thus, psychological safety could be a crucial prerequisite for interpersonal communication or fostering communication processes in difficult circumstances (O'Leary, 2016).

Especially in *obstetric care*, difficult situations that require interpersonal risk-taking and good communication are common. In this interdisciplinary and interprofessional work environment, HCWs' diverse work philosophies and backgrounds lead to different labor, processes, and teamwork concepts. Hence, communication is indispensable to bridge differences and generating a common understanding of work (Lyndon et al., 2011). Furthermore, HCWs' capacity to communicate, listen, and empathize can profoundly impact care quality and patient satisfaction (Burgener, 2017). Obstetric care is considered “safe” as patient safety incidents are less common than in other fields. Nevertheless, communication breakdowns have severe consequences, e.g., contributing to up to 72% of all perinatal deaths (Forster et al., 2006; Lippke et al., 2021). Teamwork and communication failures between obstetric HCWs could harm both the (expectant) mother and the fetus or newborn and cause high strain for HCWs and high litigation costs (Forster et al., 2006). This indicates the imperative of enhancing teamwork and communication, particularly in obstetrics as an interdisciplinary and interprofessional care unit.

Many studies have investigated the impact of *team training interventions in healthcare*. There are a variety of training programs (e.g., TeamSTEPPS, MedTeams project, Veterans Health Administration Medical Team Training program, and TeamGAINS), which enable team members and teams to improve performance and patient safety (Kolbe et al., 2013; Raemer et al., 2016). The large literature depicts that healthcare team trainings are related to improve effectiveness specifically in terms of learning, reactions, transfer, and results (e.g., organizational and patient outcomes), which demonstrated that team interventions are associated with improving safety performances (Hughes et al., 2016). For example, the well-established team training program TeamSTEPPS is related to error rate reduction and increases teamwork and communication (Parker et al., 2019). TeamSTEPPS (mainly in an emergency context) uses communication methods and tools to foster team communication, focusing on technical communication aspects (Derksen et al., 2022). Further team interventions such as TeamGAINS (Kolbe et al., 2013) were aiming to focus on a technical viewpoint and mostly investigated one single technical strategy of communication such as debriefings (Kolbe et al., 2013), speaking up (Kolbe et al., 2012; Raemer et al., 2016), or after-event reviews (and voice behavior; Weiss et al., 2017) to increase team performance perception and patient safety. Selected high-quality intervention studies in diverse health contexts that systematically examined effects on communication, coordination, or situational awareness can be found (e.g., Kolbe et al., 2013; Hughes et al., 2016; Raemer et al., 2016; Milton et al., 2023). Nevertheless, the literature shows that communication has been trained and evaluated mainly combined with other teamwork dimensions or singular technical communication skills in numerous healthcare trainings.

Looking into communication interventions specifically in obstetrics, previous studies have mixed results. The sparse research concerning interventions focusing on communication lacks clear evidence regarding underlying *psychological mechanisms* and high-quality investigations (Merién et al., 2011; Lippke et al., 2021). Moreover, most failures are based on systems rather than individuals (Derickson et al., 2015). Therefore, team interventions are suitable for reducing errors and improving team performance perception (Merién et al., 2011).

In obstetric care, team compositions and requirements in teaching hospitals alter depending on the specific context, birth situation (e.g., spontaneous birth in the delivery room, cesarean section, and emergencies), and the level of care which is prerequired. Nevertheless, there are some general insights:

The size of obstetrics teams varies, but they typically consist of several healthcare professionals, including obstetricians, midwives, nurses, anesthesiologists, and pediatricians. The peculiarity of the university hospital leads to the fact that there are continuously midwives and nurses under training and residents. Depending on the capacity of the delivery room, the staff is responsible for several births. In addition, to the care by midwives and doctors, nurses predominantly care in the ward. Obstetric teams are based on constantly adapting team structures with continuous elements of intensive and stable cooperation.

In two German university hospitals, the TeamBaby project aimed to implement communication intervention to train interdisciplinary and interprofessional team members together.

There is evidence that after a debriefing intervention (TeamGAINS), psychological safety (and leader inclusiveness) significantly increased (Kolbe et al., 2020), which could indicate that psychological safety is a crucial prerequisite for communication interventions. Against this theoretical background, the *current study* aims to draw attention to psychological safety as a crucial factor in interpersonal communication and baseline for team training. To improve psychological safety and interpersonal communication, a team intervention is developed and tested in obstetrics.

To systematize the evaluation approach, the *input–process–output model* of team effectiveness (IPO) is applied to communication, psychological safety, and perceived safety performances. The IPO is used to systematically analyze the communication team intervention to gain a comprehensive understanding of aspects which might affect individual team member's perceptions in relation to patient safety and communication. Consequently, we adapt the IPO to an individual level to investigate individual perceptions of HCWs in the context of a team intervention. The IPO is a system theory that describes how specific factors interact with each other to result in output (performance; Stewart and Barrick, 2000).

Obstetric teams are characterized as complex and by frequently changing team members due to multiple and different responsibilities, different levels of experience, and unplannable birth processes or complications. Complex and rapidly changing team characteristics are common in teaching hospitals since they must deal with all levels of risks, therefore high-risk patients, training conditions, and specializations (e.g., pelvic position birth). The IPO represents a framework that shows which important inputs are necessary to achieve outputs (Stewart and Barrick, 2000). Applied to our context, the specific obstetric team characteristics and psychological safety represent important inputs in the IPO framework that are necessary to achieve outcomes such as patient safety and team performance perception improvement.

Therefore, the research objective was to examine how psychological safety (as IPO input) fosters communication (IPO process), which leads to higher safety performance (IPO output, operationalized as perceived patient safety risks and team performance perception; Stewart and Barrick, 2000) in the context of an interdisciplinary team training (as IPO input).

In doing so, we contribute to the inconsistent teamwork and communication literature to shed new clear evidence on how and under which conditions communication interventions foster safety performance by interpreting the IPO on an individual level. The individual level of psychological safety research emphasizes the level of team members experiencing interpersonal safety or non-threat (Edmondson, 1999; Frazier et al., 2017).

Team members' perceptions and attitudes concerning teamwork are linked to patient safety and quality care (Manser, 2009; Müller et al., 2018; Kämmer et al., 2023), summarizing that the perceived teamwork quality differs depending on the profession, status, experience, or hierarchical position (Kämmer et al., 2023). Therefore, the subjective, self-perception analysis furnishes insights into social teamwork interactions, training activities, and outputs (such as patient safety risks and team performance perception) of it.

In more detail, we hypothesize the following:

**H1**: The interpersonal communication intervention increases perceived communication (H1a), perceived psychological safety (H1b), and perceived team performance perception (H1c) and decreases perceived patient safety risks (H1d).

**H2**: Perceived psychological safety at baseline is associated with less perceived patient safety risks (H2a) and higher perceived team performance perception (H2b) after the communication training.

**H3**: The association between perceived psychological safety and perceived patient safety risks (H3a), as well as perceived team performance perception (H3b) are both mediated by perceived communication.

## 2. Materials and methods

The study was conducted as part of the research project “TeamBaby – Safe, digitally supported communication in obstetrics and gynecology” (ClinicalTrials.gov Identifier: NCT03855735). The project is funded by the German Innovation Fund of the Federal Joint Committee (GBA). Lippke et al. (2019) published more specifics about the research project. The project and the used intervention were described and partially evaluated before (Derksen et al., 2022; Hüner et al., 2023). However, the aspects relating to perceived safety performances (and the psychological mechanisms) were not analyzed or published before and are unique to this manuscript.

### 2.1. Participants and procedure

Participants of the study were interprofessional team members from two German obstetric university hospitals (both perinatal center level 1^1^). The hospitals have ~2,800–3,200 deliveries per year. The sample consisted of team members who were over 18 years and who had worked at least part-time in any obstetric unit, or a gynecological unit affiliated with the delivery rooms. Physicians, midwives, nurses, healthcare workers in training, and psychologists were included in the study. Participants received information about the research project personally from on-site researchers. They obtained contact details, written information, and consent forms. The on-site researchers served as contact persons for open questions and feedback.

From January 2020 to October 2020, the HCWs were asked to answer baseline and follow-up questionnaires after the intervention, including questions regarding their communication within the team and with patients, team performance perception, perceived patient safety, and psychological safety. After that, all HCWs working in the delivery rooms were required to participate in the communication intervention described below. From March to June 2020, the training sessions were paused because of the regulations regarding the COVID-19 pandemic before the training sessions and post-intervention data collection could be resumed. Thus, the time between t1 and t2 was longer than anticipated with approximately 4 to 5 months, depending on the date of the training. The study contained only the intervention group due to both ethical concerns (providing interventions to improve patient outcomes as quickly as possible) and practical reasons (to avoid spill-over effects and to compare patient outcomes in a separate study; Hüner et al., 2023).

In total, *N* = 141 HCWs participated in the communication training. *N* = 137 voluntarily filled out a baseline (t1) and *N* = 87 the post-intervention (t2) questionnaire. Finally, t1 and t2 questionnaires from *N* = 69 individuals could be matched based on study codes. For all variables, the percentage of missing data was under 13.04%, while the baseline measurements had an average of 11.59%. As a result of the high drop-out rate and unmatchable questionnaires, 49.64% of the post-intervention scales had missing data that could not be imputed.

HCWs received a 4-h team training, focusing on interpersonal communication. Exemplary training modules were learning units regarding speaking up, closed-loop communication, perspective change, and mental models. A detailed overview of socio-demographics is provided in Table 1.

**Table 1:** Overview of socio-demographic data and experience among obstetric HCWs.

	***N* = 137**	**Physicians (*n* = 44, 32%)**	**Midwives (*n* = 43, 31%)**	**Nurses (*n* = 23, 17%)**	**Others (e.g., Trainees, Psychologist) (*n* = 22, 16%)**
Sex	Women (*n* = 122, 89%)	39 (91%)	42 (98%)	21 (91%)	19 (86%)
	Men (*n* = 10, 7%)	4 (9%)	1 (2%)	2 (9%)	3 (14%)
	Missing (*n* = 5, 4%)				
Age	< 26 years (*n* = 28, 20%)	1 (2%)	12 (29%)	3 (13%)	12 (57%)
	26–40 years (*n* = 73, 53%)	35 (85%)	20 (48%)	14 (61%)	4 (19%)
	41–55 years (*n* = 21, 15%)	4 (10%)	9 (21%)	3 (13%)	4 (19%)
	>55 years (*n* = 6, 4%)	1 (2%)	1 (2%)	3 (13%)	1 (5%)
	Missing (*n* = 9, 7%)				
Experience	< 1 year (*n* = 21, 15%)	4 (9%)	7 (17%)	5 (23%)	5 (24%)
	1–5 years (*n* = 54, 39%)	20 (47%)	19 (45%)	5 (23%)	10 (48%)
	>5 years (*n* = 54, 39%)	19 (44%)	16 (38%)	12 (55%)	6 (29%)
	Missing (*n* = 8, 6%)				

Frequencies and percentages are shown for each occupational group; percentages are in parentheses. Up to nine participants did not provide information on sex, age, and/or level of experience, and/or profession.

### 2.2. The interpersonal communication intervention

The intervention was described and partially evaluated before (Derksen et al., 2022; Hüner et al., 2023). Hughes et al. (2016) meta-analysis of healthcare team trainings indicates that healthcare team trainings must deal with specific team requirements and team characteristics such as less stability in terms of time, short team life durability, functional roles, highly different fields of competence, shared leadership, interdependence, and authority gradients. These team structures and characteristics underline the important role of communication abilities, to manage teamwork and provide safe patient care (Hughes et al., 2016). Thus, the current training is derived from the previous findings of team training research by aiming to address central interpersonal communication challenges and tools.

The interpersonal communication training was cooperatively developed by the interdisciplinary research project team (psychologists, public health experts, and obstetric HCWs) and two external communication trainers in the field of patient safety. The intervention was designed as a 4-h team training, to ensure adaptation to the stressful and time-consuming daily care routine. The trainings were conducted with the external professional trainers. Participants of the training were interprofessional and interdisciplinary team members of the obstetric units. Thus, physicians, residents, nurses, midwives, midwives and nurses under training, and psychologists were simultaneously trained in person as a group. Anesthesiologists and pediatricians were also invited to the training but did not participate. Finally, only HCWs who were directly employed in the obstetric departments participated in the intervention.

A total of 13 training sessions were performed at the two hospitals of the study, and *N* = 141 HCWs were finally trained. In the 4-h team training session, between 8 and 16 participants from all professional groups and all levels of experience participated. The intervention aimed to convey an understanding of the important role of communication in relation to patient safety and teamwork. The team training focused on combining knowledge transfer, interactive exercises, role plays, and debriefings.

Following Kolbe et al. (2020), the intervention setting and the trainer behavior guidelines were designed to establish psychological safety. The intervention sessions were placed in quiet rooms, separated from the daily work settings, and a circle of chairs was the main setup to foster interaction and discussion. The trainers were part of the circle of chairs to demonstrate being on a par. The trainers varied their positions in different exercises, e.g., they were close to participants in difficult speaking up simulations to support and reduce feelings of stress or threat. In other exercises, they were more in observational perspectives and physically further away from the participants to capture important observations or non-verbal behaviors, if necessary, and to provide feedback (Kolbe et al., 2020).

Debriefings of exercises were a central element of the training to clearly work through processes and mistakes to increase teamwork and communication, again in accordance with research showing how psychological safety can be established in healthcare debriefings (Kolbe et al., 2020). The trainers were required to create an environment that was as psychologically safe as possible so that HCWs were able to talk adequately about mistakes and improvements in the exercises. To establish psychological safety (especially at the beginning of each debriefing), the trainers explained the process and the roles of all parties involved in the debriefings (trainers and participating HCWs). All training participants were explicitly invited for participating and conducting self-reflective and discovering behavior. The trainers proactively positively marked and frequently appreciated the proactive behavior of the participants to support psychological safe actions and behavior. The trainers fostered an agreement of respectful interaction and understanding of different perspectives and opinions.

In the following, important insights into the core elements of the training are provided. The training started with an introduction to clarify expectations. “Zurich resource model”-picture postcards were used to teach an understanding of different mental models (of an optimal birth). The “Zurich resource model”-picture postcards are part of the Zurich Resource Model training, which is a proven method for the targeted motives elaboration and development for scope of action. Thus, an extraordinary feature of the Zurich Resource Model is, that in addition to conscious motives, less conscious or unconscious needs are also addressed. For this purpose, participants were invited to select images (picture postcards) that represented associations with an optimal birth, which were discussed and elaborated further on in a subsequent step in a group discussion. These individual card selection tasks and birth associations in the group discussion showed that all participants had a different idea (equated with mental models) of an optimal birth (Krause and Storch, 2006).

To introduce the importance of patient safety, communication, and teamwork (deficits), the patient safety film “Just a routine operation” was integrated in the training. The film was used to critically discuss and analyze crew resource management (CRM). The participants discussed in a group session their impressions and associations and analyzed the presented erroneous routine operation regarding CRM including centering on the role of communication, support, leadership, workload, re-evaluation of the situation for patient safety, and better teamwork. The film demonstrated an exemplary way to learn from failure (Carne et al., 2012; Mcclelland and Smith, 2016).

Furthermore, challenges of team communication, speaking up, and handovers were interactively demonstrated and trained with appropriate strategies and exercises such as Tangram, closed-loop communication, speaking up, and structured handovers (ISBAR). ISBAR is a communication framework for patient handovers by standardizing the transmission of patient information. The framework structures the communication process by giving information about introduction, situation, background, assessment, and recommendation. Therefore, ISBAR was introduced as a handover tool, to reduce communication errors.

Using Tangram exercises, interpersonal communication competencies of accuracy and clarity were trained. The Tangram exercise required one team member (the director) to verbally communicate descriptions of abstract figures to another team member (the assigner), who had to puzzle the abstract figure by not knowing the appearance of the figure (Arbuckle et al., 2000). The exercise varied successively in difficulty (e.g., at the beginning no questions are allowed, no visual support, and questions are allowed). The team tasks addressed communication challenges in clarity and accuracy and were used as an introduction to the closed-loop communication strategy to communicate more efficiently in critical task situations (Härgestam et al., 2013; Abd El-Shafy et al., 2018).

“Bad handovers” with unstructured, unimportant, insufficient information were simulated in a role play. Participants had the task of finding out the most important information about the handover. In a moderated group discussion, handover errors of the bad example were identified and discussed. Furthermore, error references to everyday handovers were used. The goal of the task was to reflect on the insufficiency of interpersonal communication as well as to address the importance of structured handovers following the ISBAR strategy (Moi et al., 2019).

The concept of speaking up was already introduced with the patient safety film “Just a routine operation,” where participants have seen and discussed a blame-free and exemplary error case showing that HCWs are frequently inhibited to speak up due to hierarchies (Pattni et al., 2019). The training offered predefined case studies of speaking up situations, to provide HCW practice under simulated conditions (role plays).

Finally, an interpersonal adaptation task based on empathy maps was part of the training to practice perspective taking (perspectives of patients, team members, and supervisors) to facilitate coping with stressful and highly complex situations. The empathy maps were applied so that different professionals systemically explored the perspective of another professional group (e.g., midwives analyzed residents, residents analyzed care, senior physicians, and mothers-to-be). The results were presented in plenary sessions across all occupational groups, and similarities and differences between the other professional groups were discussed (Cairns et al., 2021). To ensure the training modules' sustainability, a learning portfolio, reminding pocket cards, and online biweekly microteachings were provided. An overview of all training modules can be found in a study by Derksen et al. (2022).

### 2.3. Measures

We assessed self-reported data at two time points, namely the baseline (t1) and post-intervention (t2), concerning perceived psychological safety, perceived interpersonal communication within the team and patients, socio-demographic data, and safety performance indicators, which were operationalized as perceived patient safety risk and perceived team performance perception. All items were measured with a six-point Likert scale with the answer options ranging from “1” (*not at all*) to “6” (*absolutely*). All items for each construct were aggregated as mean scores.

#### 2.3.1. Psychological safety

Perceived psychological safety was measured with Edmondson's (1999) adapted four-item measure. A sample item is “Working with members of this team, my unique skills and talents are valued and utilized” (Cronbach's alpha at t1 = 0.71 and McDonald's ω at t1 = 0.73; Cronbach's alpha at t2 = 0.69 and McDonald's ω at t2 = 0.70).

#### 2.3.2. Interpersonal communication within the team and patients

Interpersonal communication was measured with Rider and Keefer's (2006) interpersonal communication competencies. HCWs of the research project discussed an initial item pool, from which a seven-item scale was developed with the sample item “We as a team take the amount of prior knowledge of the patient and how much they can understand into account.” Cronbach's alpha was at t1 = 0.85 (McDonald's ω = 0.86) and at t2 = 0.88 (McDonald's ω = 0.89).

#### 2.3.3. Patient safety risks

Safety performance indicators in terms of perceived patient safety risks were assessed as an adapted 15-item preventable adverse trigger scale. The template of the risk scale was from Keller et al. (2021), a patient-centric trigger for adverse events scale, which was adapted to HCWs. We assessed how often team members perceive patient safety risks. A sample item is “Colleagues or I had insufficient knowledge of technical equipment.” Cronbach's alpha was at t1 = 0.77 and at t2 = 0.87. McDonald's ω was reported to be 0.78 at t1 and 0.88 at t2.

#### 2.3.4. Team performance perception

We assessed safety performance indicators as perceived team performance perception. We used an adapted 3-item scale from Schaubroeck et al. (2007) with the sample items “This team gets its work done very effectively” and “My team provides quality patient care” (Cronbach's alpha at t1 = 0.78 and t2 = 0.90; McDonald's ω at t1 = 0.78 and at t2 = 0.89).

We implemented strict socio-demographic safeguards to guarantee greater anonymity and a higher response rate. Consequently, sex, age, and profession were assessed as categorical data, with the reply option “I'd rather not say” for participants who considered the provision of socio-demographic information as sensitive. Age and profession were divided in four categories correspondingly (profession: “physician,” “midwife,” “nurse,” “other”; age: “younger than or 25 years,” “26–40 years,” “41–55 years,” and “56 years or above”). Sex was measured in three groups (“men,” “women,” and “diverse”).

### 2.4. Ethics approval

Ethics approval for the data collection and training at the obstetric hospitals was granted as part of the research project's ethics approval from the two Hospital Ethics Committees. Written informed consent to participate in the study was given by all participants. HCWs voluntarily participated in the baseline and post-intervention questionnaire. Attendance at the training was mandatory.

### 2.5. Data analysis

All data analyses were conducted using IBM SPSS Version 29. Pre- and post-intervention comparisons were conducted *via t*-tests for dependent samples. In detail, *t*-tests for equality of means were used to analyze differences in pre- and post-intervention scores for perceived interpersonal communication, psychological safety, patient safety risks, and team performance perception. Associations of perceived psychological safety (t1) with perceived patient safety risks (t2) and perceived team performance perception (t2) were tested *via* multiple regression analysis. Two mediation analyses were conducted to examine the association between self-reported psychological safety (t1) and patient safety risks (t2) as well as psychological safety (t1) and team performance perception (t2) with the mediator interpersonal communication (t2). The Baron and Kenny approach was applied along with a direct test for the indirect effect *via* bootstrap analyses using 5,000 resamples by applying the Process macro model 4 for SPSS version 3.4 (Hayes, 2013).

Obstetrics is a highly diverse environment; consequently, team members have different work approaches, language use or responsibilities, and hierarchical positions (Forster et al., 2006; Okuyama et al., 2014; Schmiedhofer et al., 2021). Thus, we controlled for professional experience, age, and gender that may be associated with the HCWs' perception and communication, which were added as dummy-coded covariates. For profession, “physicians” was used as the reference group. Concerning age, “younger than or 25 years” was chosen as the reference group. Sex was included as a binary variable as no participants indicated being diverse.

As part of the retrospective Type S and M error analyses, we calculated the average of all the Type M and S errors from the observed estimates. With a statistical power of almost 81%, an average Type M error of 2.256 with a range between 1.344 and 5.042 and an average Type S error of 0.116 with a range between 0.019 and 0.260 were obtained, which means statistically significant results are on average an overestimation of 23% of the hypothesized population effect (Gelman and Carlin, 2014; Altoè et al., 2020).

## 3. Results

### 3.1. Pre-post comparison

Descriptive statistics and difference scores among variables are reported in Table 2. The changes from t1 to t2 were analyzed *via t*-tests, but there were no significant differences between the pre- and post-intervention in communication (not matching H1a), psychological safety (not supporting H1b), nor in team performance perception (not matching H1c). There was a significant difference in perceived patient safety risks (supporting H1d). On average, perceived patient safety risks were higher before (*M*_*T*1_ = 3.220, *SD*_*T*1_ = 0.735) than after the intervention (*M*_*T*2_ = 2.887, *SD*_*T*2_ = 0.902). This change with a difference score = 0.333, 95% CI [0.092, 0.573] was statistically significant (*t*(67) = 2.760, *p* = 0.007) (Table 2).

**Table 2:** Sample descriptive using *t-*test for preintervention and post-intervention equality of means.

	**Timepoint 1**			**Timepoint 2**					
**Variable scores**	***n***	***M***	***SD***	***n***	***M***	***SD***	***t(df)***	***p***	**Effect size (** ***d*** **)**
Communication behavior	68	4.64	0.68	68	4.56	0.71	0.89(67)	0.377	0.108
Psychological safety	67	4.45	0.94	67	4.51	0.83	−0.50(66)	0.617	−0.061
Patient safety risk	68	3.22	0.73	68	2.89	0.90	2.76(67)	0.007	0.335
Team performance perception	67	4.90	0.63	67	4.90	0.81	0.00(66)	1.000	0.000

*t*-tests for dependent samples and equality of means to analyze differences in pre- and post-intervention scores.

Multiple regression analysis revealed no significant association between perceived psychological safety at t1 and less perceived patient safety risks (not supporting H2a) or higher perceived team performance perception at t2 (not supporting H2b)^2^.

### 3.2. Mediation analyses

**Figure 1:**
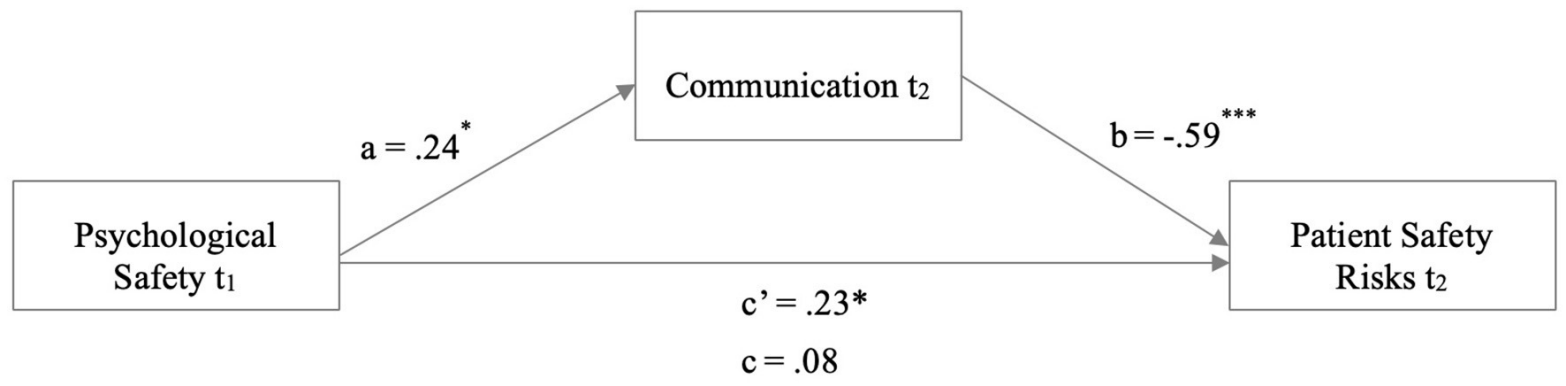
Mediation analysis with the outcome variable patient safety risks. Mediation analysis in an obstetric HCW sample. Coefficients are reported as unstandardized regression coefficients for the relationship between psychological safety and patient safety risks mediated by communication. ^*^p < 0.05 and ^***^p < 0.001.

The mediation analysis that was conducted to examine the association between self-reported psychological safety (t1) and patient safety risks (t2) with the mediator interpersonal communication (t2) only partly supported H2 and H3. Psychological safety (t1) was not associated with perceived patient safety risks (t2) directly (γ_1_′=.259, *p* = 0.038), and there was no significant total standardized effect (γ_1_= 0.096, *p* = 0.468). Psychological safety (t1) was associated with communication (t2; α_1_= 0.329, *p* = 0.013). Furthermore, communication (t2) was significantly associated with patient safety risks (t2; β_1_ = −0.497, *p* < 0.001; Figure 1). Lastly, bootstrapping procedures using 5,000 resamples revealed a significant standardized indirect effect of psychological safety (t1) on patient safety risks (t2) mediated by communication (t2; $\alpha_{1}^{*}\beta_{1}=-$0.163, 95% CI [−0.310, −0.046]). Overall, 31.6% of the risk's variance could be explained with psychological safety and communication thereby.

**Figure 2:**
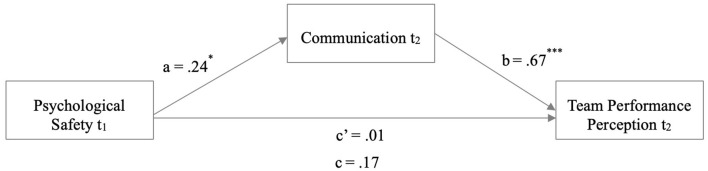
Mediation analysis with the outcome variable team performance perception. Mediation analysis in an obstetric HCW sample. Coefficients are reported as unstandardized regression coefficients for the relationship between communication and team performance perception mediated by psychological safety. ^*^p < 0.05 and ^***^ *p* < 0.001.

The mediation analyses examining the association between psychological safety (t1) and team performance perception (t2) with the mediator interpersonal communication (t2) also did not reveal the hypothesized direct effects but again showed a significant indirect effect. Psychological safety (t1) was not associated with team performance perception (t2) directly (γ_2_′= 0.010, *p* = 0.931), and there was no significant total standardized effect (γ_2_= 0.200, *p* = 0.141). Psychological safety (t1) was associated with communication (t2; α_2_= 0.329, *p* = 0.012). Furthermore, communication (t2) was significantly associated with team performance perception (t2; β_2_=.574, *p* < 0.001; Figure 2). Lastly, bootstrapping procedures using 5,000 resamples revealed a significant standardized indirect effect of psychological safety (t1) on team performance perception (t2) mediated by communication (t2; $\alpha_{2}^{*}\beta_{2}=$ 0.189, 95% CI [0.044, 0.370]).

Overall, 34.0% of the team performance perception's variance could be explained with psychological safety and communication thereby.

## 4. Discussion

The current study's aim was to examine psychological mechanisms of a communication team training to increase patient safety and team performance perception, as well as psychological safety as an important antecedent of interpersonal communication. The present research illustrates that communication is crucial for safety performance as a mediating factor in healthcare teams such as obstetrics.

Surprisingly, contrary to our assumptions, there were no significant pre- and post-differences before versus after the training in interpersonal communication, psychological safety, nor team performance perception. This speaks for rather stable, resisting patterns and little change over time. However, as predicted, perceived patient safety risks decreased post-training. Regarding interpersonal communication, psychological safety, and team performance perception, HCW's already high scores at the first time point could be attributed to several biases, such as social desirability (Chung and Monroe, 2003), a ceiling effect (Wang et al., 2009), or the better-than-average-effect that describes the propensity to rate oneself better than others, e.g., in behavior or norms (Alicke et al., 1995; Sedikides et al., 2005). Regarding the ceiling effect, HCWs already considered their perceived interpersonal communication as very high before the training. This could have been a biased assessment, but it also could reflect actual high standards in the university hospitals. Accordingly, no decrease can also be seen as an advantage, especially as the stable pattern can be attributed to the intervention but also just a contextual effect as no control group was used as a comparator. As part of the communication team training, participants learned and dealt with challenges and misassumptions of interpersonal communication embedded in teamwork scenarios, which may lead to a higher reflection of their own and team (communication) competencies (Koole et al., 2011). Hence, it is likely that the assessment of the team and own skills became more critical after the intervention.

The communication intervention was designed as a 4-h training. Steinemann et al. (2011) demonstrated a 4-h concept of team training, which was associated with improved teamwork and clinical performance for multidisciplinary trauma teams. Emerging from this study, we conclude that the brief intervention time is suitable to maintain patient safety and team performance perception. To achieve a successful transfer into the daily work routine, HCWs require specific conditions to train under time-critical, stressful, and complex simulations (Hughes et al., 2016); therefore, all training modules and debriefings varied in conditions and difficulty levels.

The training aimed to address central communication challenges. The participants were educated about non-congruence of individuals' thought worlds (mental models), for which intersections can be established through communication. Mental models are mental representations, which capture an individual's understanding of a particular area in their mind. They are an essential concept for organizations and teamwork to improve learning since the understanding of how information is constructed and how individuals behave requires the use of mental models. The training provided the introduction and discussion of mental models in relation to communication (Rook, 2013). Therefore, the training focused on communication processes to create a shared understanding (Verdonik, 2010). The Tangram exercise aimed to practice the quality of interpersonal communication. Clarity is the degree to which interpersonal communication avoids purposeful or unintentional vagueness, ambiguity, and ambiguous language, as opposed to communication accuracy, which relates to the correctness of transmitted message contents (e.g., validity of information; Hannawa et al., 2017). The Tangram exercises depicted communication challenges and aimed to facilitate communication more accurately and sufficiently.

The “Zurich resource model”-picture postcards are in line with psychotherapeutic assumptions that individuals have most of the resources to solve problems within themselves. The postcards contain images that triggered positive feelings (Krause and Storch, 2006). Therefore, the training aimed to induce positive resources (positive associations of birth), which have translated into communication to be shared with other team members and professional groups to gain insights into differences and commonalities. The discussion of the picture postcards, which were associated with an optimal birth, led to the realization that everyone has different ideas of birth. Thus, differences in mental models came into language and hence shared mental models could come into being, which is in line with organizational learning eventually appears through individual members (Rook, 2013).

Furthermore, different from previous expectations, no significant associations between perceived psychological safety (t1) and decreased patient safety risks (t2), as well as increased team performance perception (t2) after the training, could be found. Research depicts the maladaptation of healthcare organizations by suffering from stiff, profession-based hierarchies, hindered open error discussions, and tendencies to blame individuals instead of understanding errors as system-generated (Tucker et al., 2007). To counteract these problematic factors, research has illustrated psychological safety as crucial for such demanding work structures as it ensures high-quality care and patient safety (O'Donovan et al., 2021). Adding to this literature, the current study empirically demonstrates psychological safety as a fundament of safe communication that, in turn, can improve patient safety and team performance perception. Thus, the study provides further guidance on how to deal with difficult teamwork and structural challenges in the healthcare system.

Moreover, it has been shown that applying the ISBAR strategy in handovers is related to increased patient safety, interprofessional teamwork, awareness of communication (errors), and professional roles (Haddeland et al., 2022). The intervention simulated teamwork and communication challenges by handovers and introduced ISBAR as structured handover tool. Despite existing handover guidelines at both hospitals, the background information about the importance of standardization and structurization of handovers was well-received as fostering patient safety.

Current literature points out that psychological safety supports interpersonal communication, which is required for teamwork and patient safety (Lei, 2014; Jain et al., 2016). Nevertheless, there are inconsistencies in the direction of the association between psychological safety and communication (Siemsen et al., 2009). Studies show that if psychological safety is lacking, patients and healthcare providers interfere with effective care by withholding important information (patients' information, e.g., ambiguity or HCWs knowledge, e.g., research findings; Jain et al., 2016). Moreover, psychologically safe teams tend to discuss more freely with fewer boundaries and risk of being blamed (more voice and speaking up behavior; O'Donovan et al., 2021). Although the advantages of increasing psychological safety within healthcare teams have been demonstrated, interventions are needed to implement these in daily care (O'Donovan and McAuliffe, 2020).

Our study meets O'Donovan and McAuliffe (2020)'s call for interventional needs and practical implementation by implicating that psychological safety predicts communication. Our mediation model revealed that psychological safety as input is only associated with patient safety and team performance perception as output through communication as an intervening mechanism, which is further validated by the IPO model (Stewart and Barrick, 2000).

Effective communication has been broadly found to be positively linked to improved individual, team, and organizational performance. In healthcare, communication is associated with higher patient and HCW's satisfaction, learning, collaboration, and performance outcomes (Jain et al., 2016; O'Donovan and McAuliffe, 2020). Communication errors are primarily discovered in hierarchical conflicts as well as interpersonal conflicts and power issues, thus reflecting poor psychological safety (Yanchus et al., 2014).

Therefore, speaking up was introduced to educate the competence to raise concerns and challenge authority for safety reasons. Speaking up is essential to improve patient safety; nevertheless, it is difficult to speak up due to fear of negative consequences (e.g., career loss and job difficulties), fear of rejection, or disciplinary consequences (Okuyama et al., 2014). Consequently, the training addressed authority gradients and how to deal and communicate errors by practicing speaking up situations in a psychological safe case simulation. In psychologically safe environments, employees described better interpersonal communication and had a higher level of feeling more secure in speaking up, asking questions, and exchanging ideas (Yanchus et al., 2014; O'Donovan and McAuliffe, 2020). Thus, psychological safety fosters an atmosphere that helps team members communicate safer to prevent errors and increase teamwork due to higher team performance perception. To address challenges, our interdisciplinary and interprofessional communication training simulated these difficult interpersonal situations and introduced specific communication strategies such as empathy maps or shared understanding.

As already described before, psychological safety supports patient safety, collaboration, learning from mistakes, and adverse events (Hirak et al., 2012; Arnetz et al., 2019), as well as speaking up behavior or the reduction in silence behavior (Newman et al., 2017). Therefore, in line with Kolbe et al. (2020), psychological safety is an essential requirement for efficient debriefings. We regard the built-in debriefings in the training as fundamental to train and improve communication and handling mistakes in a psychological safe training environment.

The empathy map training element elaborated the other professional and patient perspective about (work) tasks, feelings, thoughts, and fears. Empathy includes the ability to understand other perspective (e.g., of patients or colleagues) and to communicate the individual understanding which could lead to a shared understanding (Cairns et al., 2021). The exercise frequently showed conflict potential between the professional groups by not feeling adequately represented. Nevertheless, empathizing with another professional group, sharing similarities (e.g., common goals and fears), and differences were brought into communication which could support an understanding of another's perspective. The empathy map exercise can be related to establishing psychological safety by training to respect other perspectives.

In sum, challenges of team communication, speaking up, and handovers were interactively demonstrated and trained with appropriate strategies and exercises such as tangram, closed-loop communication, speaking up, structured handovers (ISBAR), and debriefings. Thus, the training aimed to challenge and train effective communication under psychologically safe conditions to address misassumption of communication and how to generate a shared understanding of each other's (team members and patients) thoughts, feelings, and meanings to enhance communication interactions to increase patient safety (Hannawa et al., 2017).

### 4.1. Limitations of the current research and suggestions for future studies

There are a few limitations that must be considered while interpreting the results. First, no randomized-controlled trial with a control group was implemented to ensure all patients' safety. The reasons for which no control group was realized in this study were two-fold: First, we aimed to provide the intervention to all healthcare workers as quickly as possible so that team communication could be improved, and more birthing persons would benefit (ethical reasons). Other reasons were more practical, including the anticipated rather small sample size and potential spill-over effects compromising the study design, as well as the need to compare clinical routine data before and after the intervention to establish effects on clinical outcomes. The intervention was part of a larger communication project targeting healthcare workers and pregnant women from both psychological and medical perspectives so that clinical outcomes were investigated in different publications (e.g., Hüner et al., 2023). Nevertheless, changes in communication behavior from t1 and t2 should not be interpreted in terms of intervention effects as alternative explanations could account for improvements and causality cannot be established.

While analyzing HCWs' ratings, it is important to be aware of the limits of self-reported measurements, such as social desirability. The lack of validated scales in prior research led to the necessity to newly develop or modify scales. Therefore, several proposed measures have lower reliability, which must be considered a weakness of the study. Another potential risk of the self-reported scales in this setting is the risk of common source bias, potentially leading to less reliable results than objective indicators. Nevertheless, data were collected at two hospitals from team members with a wide range of characteristics, such as professional occupation, age, and experience, as well as main operational areas (e.g., postpartum units, delivery rooms, and surgical theater) and responsibilities to reduce common source bias.

There is also literature showing that perceptions of performance differ from the actual performance (e.g., Kruger and Dunning, 1999). Observational studies, objective data monitoring, or qualitative interviews, as well as an RCT design, could have helped to understand intervention effects. Over the scope of the research project, clinical routine data were analyzed comparing a time frame after the training with a time frame before the intervention (Hüner et al., 2023). Nevertheless, understanding subjective perceptions is crucial for comprehending shared work reality and mental representations (e.g., regarding psychological safety). Future studies should combine validated measures with more objective and change-sensitive measures such as incident reporting systems, routine data analysis, or patient assessments, introducing a control group and mixed-method approaches. As it was not possible to link perceptions of performance with objective team performance in this study, future research is required.

During the study course, the COVID-19 pandemic influenced the implementation of the intervention. Therefore, the presence and accompanying restrictions of COVID-19 must be considered while interpreting the findings. Our trainings were interrupted; thus, there were longer time lags of 4 to 5 months between the training and surveys. Hence, immediate changes might not have been captured. On the contrary, more long-term training mechanisms might have been uncovered which has been a challenge in previous research. HCWs were confronted with unpredictable threats, fear of infection, psychological stress, and heavy workload (Uzun et al., 2020). For example, face masks and social distancing were important protection activities; however, face masks have greatly impacted communication by muffling noise, reducing facial expressions, and creating distance (Mheidly et al., 2020). These burdens may have affected the assessments and interventions. As an alternative explanation, the intervention and surveys could have offered a reflection and learning platform of interpersonal communication and teamwork, which could have helped HCWs to better cope with the negative consequences of COVID-19. More frequent time points of measurement, including a follow-up and taking team structure into account when conducting analyses, would have been required to capture all changes in communication, but they were not possible to implement.

The high drop-out rate and small sample size could be related to the additional burden of the pandemic and the specifications of the teaching hospitals that may have prevented HCWs from participating in data collection. The results from this study may only be generalizable to interpersonal communication in obstetrics due to the relatively small sample size; other healthcare sectors need to be addressed. Future research designs could work with more follow-up measures in randomized-controlled trials to broaden our understanding of changes over time.

### 4.2. Implications for practice

According to our findings, it can be concluded that psychological safety is the initial input variable to train HCWs' interpersonal communication skills to foster patient safety and team performance perception. It can be seen that there is a lack of interventions aiming to improve psychological safety in healthcare teams and precise, objective measurements to identify when psychological safety is low and to monitor changes over time (O'Donovan and McAuliffe, 2020). Therefore, our intervention can be used as a template to design further studies on psychological safety and communication in healthcare teams. The length of our training (4 h) guaranteed an integration into the daily routine; nevertheless, longer and more intensive interventions could increase long-term effects. Larger samples should be targeted to counteract higher drop-out rates.

The implementation of health services research into everyday healthcare is associated with great challenges and resembles change processes which are often met with criticism and resistance. Further studies should ensure that internal staff with leadership functions are involved in the implementation process so that the project can be successfully implemented (Kumar, 2013). In addition, the organizational level should be incorporated ideally with a co-creative approach to ensure sustainability of the effects.

The healthcare system has no tolerance for errors; paradoxically, human mistakes are unavoidable. The medical system does not adequately educate HCWs because technical skills and examination techniques are often addressed, but handling errors and teamwork is not trained (enough). For example, physicians are seen as principal decision-makers, which neglects a system approach of a team decision process. Therefore, our communication training in a teamwork setting is indispensable filling the gap to deal with errors adequately (Robertson and Long, 2018). The introduction of systemic trainings for professionals and HCWs under training is important to bring sustainable system transformations aiming at patient safety and teamwork. The creation of expert positions dealing with social skills and system thinking in hospitals could lead to fast and efficient handling of human errors to increase the quality of care and relieve teams. The training manual can be accessed and used for free (German language).

## 5. Conclusion

Given the difficulty of patient care and different human competence problems, such as frequent communication breakdowns, can result in unintended patient harm. High-quality care and patient safety require effective teamwork and communication. To meet these requirements, our interpersonal and interprofessional team training represents a novel approach as it brings together interpersonal communication and psychological safety in the context of patient safety although the effects still need to be researched further.

In sum, our study results underscore that psychological safety may have positive effects on perceived team performance perception and inhibiting effects on perceived patient safety risk. These effects appear mediated by interpersonal communication. The reported data are embedded in the IPO model of team effectiveness underlining the psychological mechanism. Our research model displays teamwork and team complexities in healthcare by indicating communication as fruitful intervening mechanism in a psychological safe training environment to promote patient safety and team performance perception.

## Data availability statement

The data for this study are not publicly available due to data protection guidelines. The data are available on request from the corresponding author.

## Ethics statement

The studies involving human participants were reviewed and approved by Ethics Committee at Jacobs University Bremen (dated September 17, 2019); University Hospital of Ulm Human Research Ethics Committee (Number 114/19); University Hospital of Frankfurt Medical Research Ethics Committee (Number 19–292). The patients/participants provided their written informed consent to participate in this study.

## Author contributions

JED contributed to the conceptualization and study design, data collection, statistical analysis, interpretation of the analyses, and wrote the first draft of the manuscript. CD supervised and managed the data collection, supervised the analyses, and reviewed sections of the manuscript. FMK contributed to the statistical analysis, interpretation of the analyses, and reviewed sections of the manuscript. SL contributed to conception and design of the study, supervised the data collection, analyses, and reviewed the manuscript. All authors contributed to the article and approved the submitted version.

## Footnotes

^1^The participating HCWs of the hospitals were part of numerous obstetric teams with a very dynamic, always changing composition. These obstetric teams were composed based on individual care structure and indications. Birth processes in the participating hospitals (high risk and high complication rates) are often unpredictable; thus, the obstetric teams need to adapt to individual birth conditions. On a daily basis in the delivery room, approximately four to five team members (midwives, physicians, and pediatricians) are assigned to one patient/expected mother. In other contexts, such as emergencies or cesarean operations, the teams consist of an operating nurse, a pediatrician, physician, a midwife, and an anesthetist. In the ward, primarily nurses, midwives, and ward physicians are with the patients. Team members hand over their patients several times a day within the occupational groups; here, the respective occupational groups meet for patient handover and discuss the processes and cases. In general, midwives and obstetricians have very intensive and stable cooperation over time, and pediatricians and anesthesiologists are temporarily and partially integrated into the care process as needed. As depicted, the participating HCWs are part of constantly adapting team structures with continuum elements. All employees at the participating obstetric clinic feel as one big team. They have common team meetings, scientific workshops, trainings, debriefings, and feedback rounds, as well as joint further education, simulation practices and shared breaks, break rooms, shared goals, or shared activities.

^2^While analyzing the participating obstetric units separately in multiple regression analyses, no differences were seen across the clinics. The results revealed no significant association between perceived psychological safety at t1 and less perceived patient safety risks (not supporting H2a) or higher perceived team performance perception at t2 (not supporting H2b) for both participating obstetric units.
