# Supplementary material for: Interdisciplinary and interprofessional communication intervention: How psychological safety fosters communication and increases patient safety
Source: Front Psychol. 2023 Jun 15;14:1164288. doi: 10.3389/fpsyg.2023.1164288 (PMC10310961; doi:10.3389/fpsyg.2023.1164288)
Supplement: Supplementary file 1 [file Table_1.DOCX]

Supplementary Material

Interdisciplinary and interprofessional communication intervention: How psychological safety fosters communication and increases patient safety

Johanna Elisa Dietl^*^, Christina Derksen, Franziska Maria Keller, Sonia Lippke

*** Correspondence:** Johanna Elisa Dietl: jdietl@constructor.university

# Supplementary Tables with translated measures (used items were worded in German)

## Table S1: Measurement of Psychological safety

| Scale Introduction | Item |
| --- | --- |
| Please think more in detail about your team: How much do the following statements apply to you? | Working with members of my team, my unique skills and talents are valued and utilized. |
|  | Members of my team are able to bring up problems and tough issues. |
|  | No one on my team would deliberately act in a way that undermines my efforts. |
|  | It is difficult to ask other members of my team for help. |

*Note.* All items were measured with a six-point Likert scale with the answer options ‘1’ (*not at all*) ranging to ‘6’ (*absolutely*). Adapted scale by Edmondson, A. C. (1999). Psychological safety and learning behavior in work teams. *Administrative Science Quarterly, 44*(2), 350–383.

## Table S2: Measurement of Interpersonal communication within the team and patients

| Scale Introduction | Item |
| --- | --- |
| We as a team …  *“We” means you and your colleagues, i.e. those with whom you normally work in a team (not just your own professional group).* | …answer all patients' questions sufficiently. |
|  | … explain treatments or procedures, in such a way that we are sure that the explanation is completely correct. |
|  | … explain all examinations or procedures in such a way that the patients understand them. |
|  | … also involve the people accompanying the patients in decisions. |
|  | … listen to the patients' concerns and fears. |
|  | … consider how much prior knowledge a patient has and how much they can understand. |
|  | … can deal well with language barriers on the part of patients and accompanying persons in our everyday work. |

*Note.* All items were measured with a six-point Likert scale with the answer options ‘1’ (*not at all*) ranging to ‘6’ (*absolutely*). Newly devised scale based on Rider, E. A., & Keefer, C. H. (2006). Communication skills competencies: Definitions and a teaching toolbox. *Medical Education, 40*(7), 624–629. <https://doi.org/10.1111/j.1365-2929.2006.02500.x>

## Table S3: Measurement of Patient safety risks

| Scale Introduction | Item |
| --- | --- |
| On a routine working day, I usually notice that... | ... colleagues have poorly washed or disinfected their hands. |
|  | ... there were conflicts among colleagues. |
|  | ... patients and their companions were poorly informed about examinations and treatment measures. |
|  | ... generally applicable duties of care were ignored. |
|  | ... colleagues forgot to pass on important information. |
|  | ... my opinions and suggestions were ignored. |
|  | ... important information (e.g. about allergies) was missing from a patient file. |
|  | ... processes did not work smoothly. |
|  | ... colleagues or I myself were emotionally burdened. |
|  | ... colleagues or I myself were not familiar enough with technical equipment. |
|  | ... colleagues or I myself were poorly prepared for conversations with patients and their companions. |
|  | ... too few important or useful treatment options (e.g. medication, physiotherapy, etc.) were taken into account. |
|  | ... important information was missing from discharge reports. |
|  | ... the cooperation between the hospital and external treatment providers did not work well. |
|  | … conflicts arose due to language barriers. |

*Note.* All items were measured with a six-point Likert scale with the answer options ‘1’ (*not at all*) ranging to ‘6’ (*absolutely*). Adapted scale by Keller, F. M., Derksen, C., Kötting, L., Schmiedhofer, M., & Lippke, S. (2021). Development of the perceptions of preventable adverse events assessment tool (PPAEAT): measurement properties and patients’ mental health status. *International Journal for Quality in Health Care*, *33*(2)

## Table S4: Measurement Team performance indicators.

| Scale Introduction | Item |
| --- | --- |
| In general, how would you rate the performance of your interdisciplinary team? | This team is very competent. |
|  | This team gets its work done very effectively. |
|  | My team provides quality patient care. |

*Note.* All items were measured with a six-point Likert scale with the answer options ‘1’ (*not at all*) ranging to ‘6’ (*absolutely*). Adapted scale by Schaubroeck, J., Lam, S. S., & Cha, S. E. (2007). Embracing transformational leadership: Team values and the impact of leader behavior on team performance. *Journal of applied psychology*, *92*(4), 1020.
